# RNA-seq analysis and fluorescence imaging of melon powdery mildew disease reveal an orchestrated reprogramming of host physiology

**DOI:** 10.1038/s41598-019-44443-5

**Published:** 2019-05-28

**Authors:** Álvaro Polonio, Mónica Pineda, Rocío Bautista, Jesús Martínez-Cruz, María Luisa Pérez-Bueno, Matilde Barón, Alejandro Pérez-García

**Affiliations:** 1 Departamento de Microbiología, Facultad de Ciencias, Universidad de Málaga, Bulevar Louis Pasteur 31, 29071 Málaga, Spain; 2 Instituto de Hortofruticultura Subtropical y Mediterránea “La Mayora”, Universidad de Málaga, Consejo Superior de Investigaciones Científicas (IHSM−UMA−CSIC), Bulevar Louis Pasteur 31, 29071 Málaga, Spain; 3 Departamento de Bioquímica y Biología Celular y Molecular de Plantas, Estación Experimental del Zaidín, Consejo Superior de Investigaciones Científicas (CSIC), Profesor Albareda 1, 18008 Granada, Spain; 4 Plataforma Andaluza de Bioinformática, Edificio de Bioinnovación, Severo Ochoa 34, Parque Tecnológico de Andalucía, 29590 Málaga, Spain

**Keywords:** Biotic, Secondary metabolism

## Abstract

The cucurbit powdery mildew elicited by *Podosphaera xanthii* is one of the most important limiting factors in cucurbit production. Our knowledge of the genetic and molecular bases underlying the physiological processes governing this disease is very limited. We used RNA-sequencing to identify differentially expressed genes in leaves of *Cucumis melo* upon inoculation with *P*. *xanthii*, using RNA samples obtained at different time points during the early stages of infection and their corresponding uninfected controls. In parallel, melon plants were phenotypically characterized using imaging techniques. We found a high number of differentially expressed genes (DEGs) in infected plants, which allowed for the identification of many plant processes that were dysregulated by the infection. Among those, genes involved in photosynthesis and related processes were found to be upregulated, whereas genes involved in secondary metabolism pathways, such as phenylpropanoid biosynthesis, were downregulated. These changes in gene expression could be functionally validated by chlorophyll fluorescence imaging and blue-green fluorescence imaging analyses, which corroborated the alterations in photosynthetic activity and the suppression of phenolic compound biosynthesis. The powdery mildew disease in melon is a consequence of a complex and multifaceted process that involves the dysregulation of many plant pathways such as primary and secondary metabolism.

## Introduction

The Cucurbitaceae or cucurbit family includes many economically important species, particularly those with edible fruits such as cucumber, melon, watermelon, zucchini and pumpkin^1^. Unfortunately, phytopathogens hinder the production of cucurbits, leading to over 200 known diseases with diverse aetiologies^2^. Cucurbit powdery mildew is the primary fungal disease that affects cucurbits under both greenhouse and open field conditions. Like other powdery mildew diseases, it is easily recognizable by characteristic symptoms such as the whitish, talcum-like, powdery fungal growth that develops on leaf surfaces, petioles and stems, and rarely on fruits^2,3^. There are two species that can cause powdery mildew in cucurbits around the world, namely, *Golovinomyces orontii* and *Podosphaera xanthii*. The latter is considered to be the main causal agent of powdery mildew in cucurbits, and it is one the most important limiting factors for cucurbits production^4–6^.

The arrival of a conidium to the leaf of a susceptible host and its adhesion, penetration, nutrition and proliferation as well as plant defence response suppression, are necessary steps for the establishment of a compatible interaction^7^. Like most powdery mildew fungi, *P*. *xanthii* grows on the foliar surface, taking nutrients from the epidermal host cells through the development of a specialized parasitism-related structure called the haustorium^8^. The epiphytic transcriptome of *P*. *xanthii* is available^9^, several molecular tools have been developed for the specific functional analysis of *P*. *xanthii* genes such as host-induced gene silencing and transient transformation^10,11^ and the transcriptomes and genomes of major crops such as cucumber, melon, watermelon and zucchini are also available^12–18^. However, the genetic and molecular bases of the physiological processes governing this intimate plant-fungus interaction remain largely unknown.

RNA-seq is a revolutionary tool for transcriptomics which has altered our view of the extent and complexity of eukaryotic transcriptomes^19^. RNA-seq analysis has been demonstrated to be an effective approach to decipher the primary changes in gene expression, providing a far more precise measurement of transcript levels and their isoforms than other methods and allowing researchers to detect transcripts with low abundance^20,21^. Fungal diseases of plants remain a major challenge in agriculture. Hence, an understanding of disease mechanisms at the molecular level is of paramount importance for identifying possible intervention points for their control^22^. For this reason, several studies have used RNA-seq analysis to obtain a more comprehensive view of the primary molecular mechanisms that are dysregulated in plants upon pathogen inoculation^23–27^. However, whole-transcriptome changes during early disease stages in susceptible plant species are less well-documented than those of resistant ones. This is the case for cucurbit-powdery mildew interactions. There are only two reports that describe the transcriptome profiles of pumpkin and melon lines resistant to powdery mildew and their comparison with susceptible cultivars^23,28^.

Different imaging techniques are currently widely used in plant physiology to assess the impact of biotic stress on host plants since they reveal the metabolic gradients that pathogens usually induce in infected leaves^29,30^. Examples of these techniques include chlorophyll fluorescence imaging (Chl-FI) and multicolour fluorescence imaging (MCFI). Studying the red chlorophyll fluorescence (Chl-F) emitted by photosystem II (PSII) provides information on the photosynthetic performance of plants in terms of activity^31^ and indirect information on the CO_2_ assimilation rate^32^. Similarly, MCFI is a very useful technique for monitoring the plant health status. It is based on recording the blue (F440), green (F520), red (F680) and far red (F740) fluorescence that leaves emit when they are excited with UV light. Particularly, the so-called blue-green fluorescence (BGF) is a valuable technique to study secondary metabolism, since phenolic compounds from the phenylpropanoid pathway are the primary emitters of that fluorescence^33,34^. To date, many plant-pathogen interactions have been subject to analysis using imaging techniques^29,35^ and most of them have the primary aim of identifying specific disease identity marks.

This study is intended to elucidate the primary alterations that take place in the physiology and metabolism *P*. *xanthii*-inoculated melon plants during the first stages of infection. For this purpose, RNA-seq analysis was used to search for changes in leaf gene expression after pathogen inoculation. In parallel, plants were phenotypically characterized by different imaging techniques to provide experimental support for the physiological changes anticipated by RNA-seq analysis. Our findings show that photosynthesis and secondary metabolism are the primary physiological processes of the host that are altered during the initial infection stages. This study is the first step in looking for effectors of *P*. *xanthii* specifically aimed at manipulating these central functions of the host physiology. Unraveling these mechanisms would be desirable as a preliminary step in the development of new melon varieties that are resistant to *P*. *xanthii*.

## Results

### Visualization of the development of *P*. *xanthii* structures during the first stages of infection

**Figure 1:**
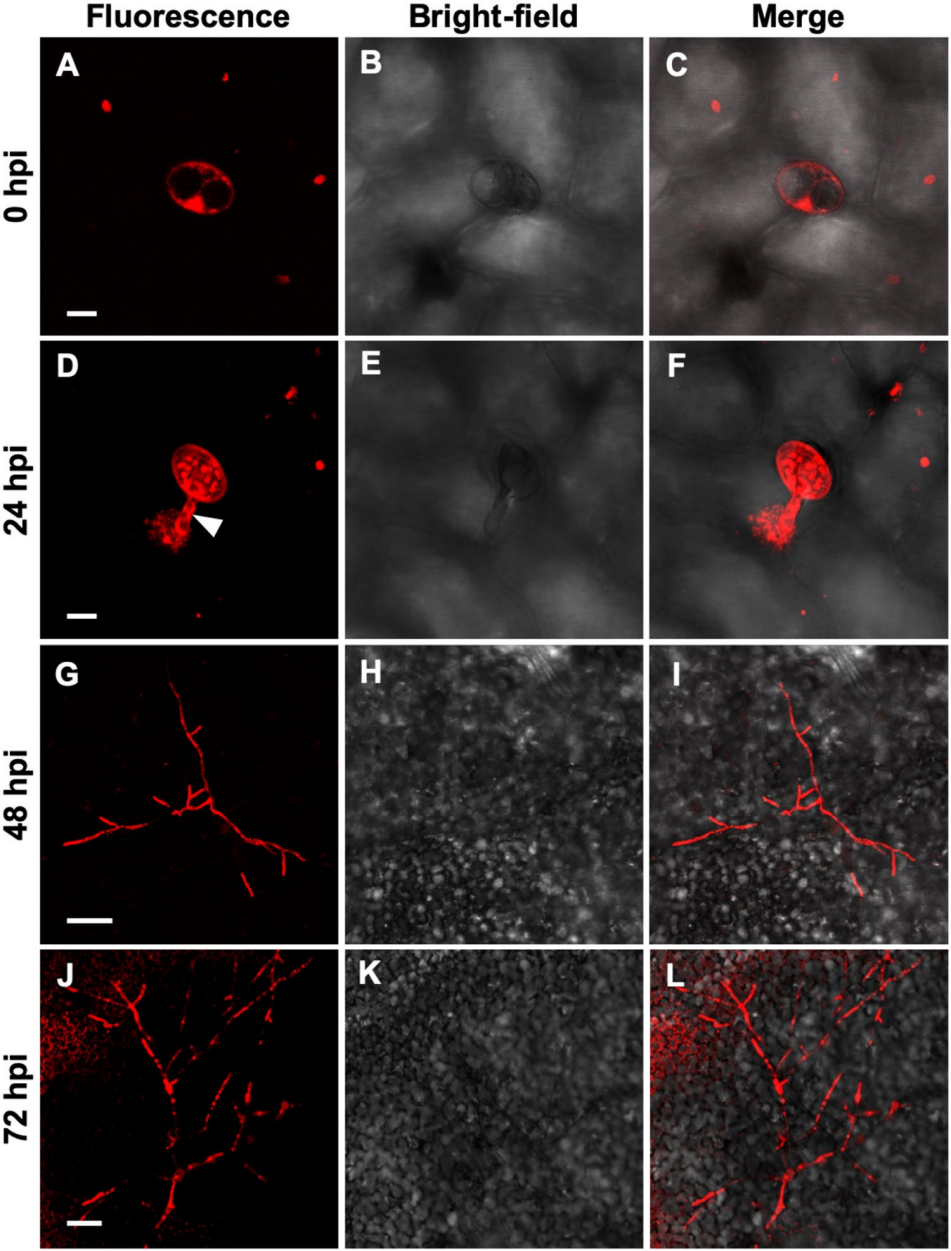
Time-course analysis on the development of *P*. *xanthii* on the leaves of melon plants used for RNA-seq analysis by CLSM. Fungal structures were stained with propidium iodide. The pictures were taken at 0, 24, 48 and 72 h post-inoculation (hpi). (**A**–**C**), an un-germinated conidium on the leaf surface. (**D**,**F**), a conidium with a germ tube (arrowhead). (**G**–**I**), a germinated spore (arrowhead) with primary hyphae and initial development of secondary hyphae. (**J**–**L**), Weft of secondary hyphae. Bars: (**A**–**F**) 10 µm; (**G**–**L**) 100 µm.

In parallel with the sampling of plant material for RNA-seq analysis, leaf disks were collected to visualize the development of *P*. *xanthii* infection structures during the period of time analyzed by RNA-seq, that is, the first 72 h of interaction (Fig. 1). At 0 hpi (hours post-inoculation), only the conidia were visible. At 24 hpi, most of the spores had germinated. At this stage, although not observed in the pictures, the first haustoria had already developed^8^. At 48 hpi, the primary hyphae were very abundant, and the initial formation of secondary hyphae could also be detected. Finally, at 72 hpi, extensive branching was observed in the secondary hypha.

### Illumina sequencing, mapping and identification of DEGs

**Figure 2:**
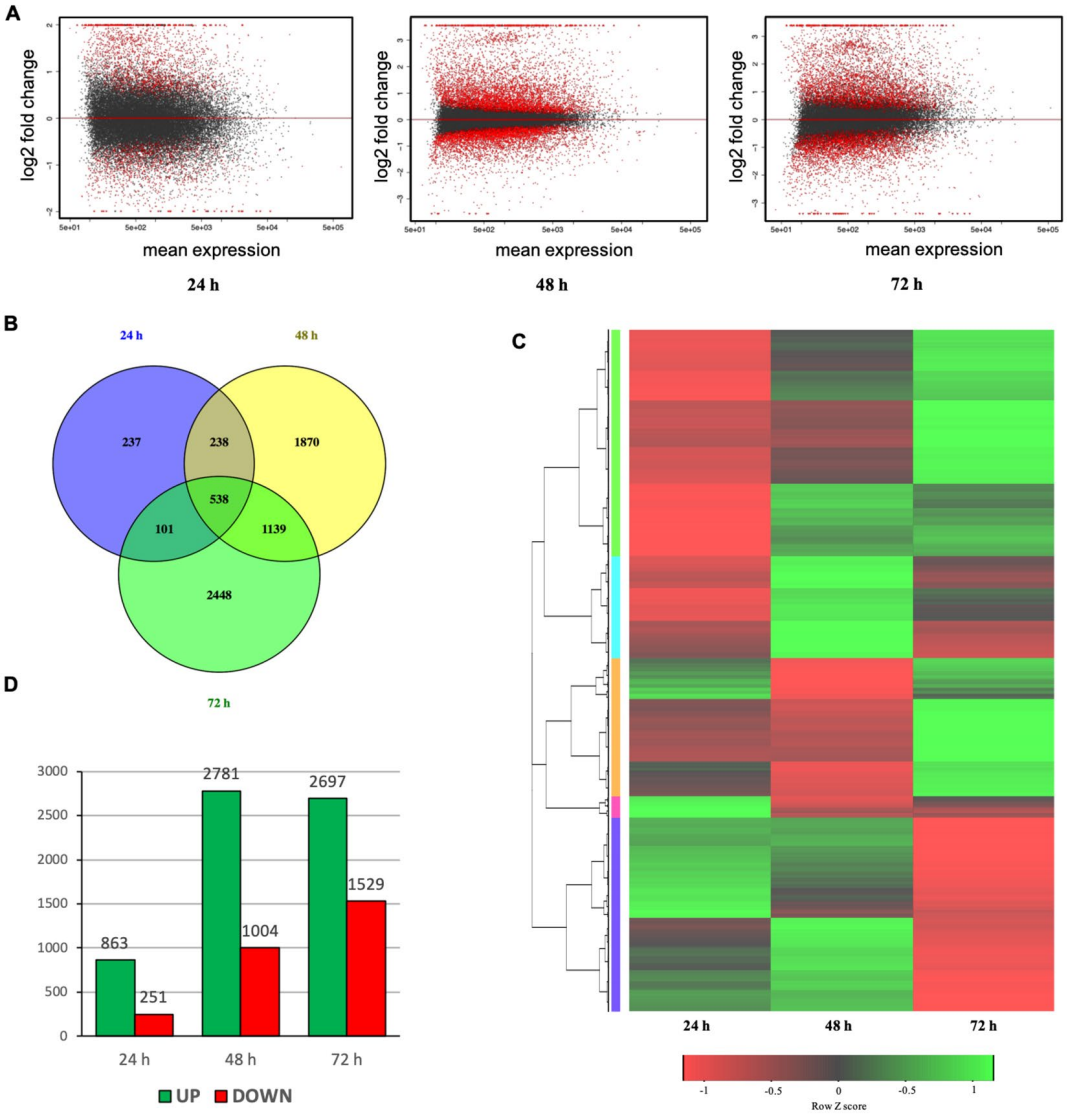
Summary of sequence data and the number of differentially expressed genes determined by RNA-seq in melon plants infected with *P*. *xanthii* versus non-infected control plants. Data were collected at 24, 48 and 72 h post-inoculation (hpi). (**A**) log2-fold change versus a mean expression scatter plot of the RNA-seq results. (**B**) A Venn diagram displaying the distribution of DEGs (genes with >1 or > −1 log2-fold change in expression) at each time point. (**C**) Hierarchical clustering of DEGS at each time point. Rows are clustered using distance and average linkage. Changes of gene expression are displayed from red (lower expression) to green (higher expression). (**D**) Number of genes up- and downregulated at each time point in infected plants compared to non-infected control plants.

High-throughput sequencing produced a total of 1661.7 million short reads, of which 86.41% (1435.9 million) passed the quality control thresholds. To calculate the expression profile, clean reads were mapped onto the melon reference transcriptome with the Bowtie2 algorithm. The alignment results were reported in SAM/BAM format. The mapping rates for various samples are summarized in Table S1. The expression estimates are reported in total counts for each gene. DESeq. 2 software was employed to analyse the differentially expressed genes at three time points (24, 48 and 72 hpi) in infected plants relative to un-inoculated control plants. Differentially Expressed Genes (DEGs) were reported in log2FoldChange (FC) with a corresponding *p-value* for each gene. An MA plot was used to represent the DEGs of infected plants relative to control plants at 24, 48 and 72 hpi (Fig. 2A). Among all the DEGs, 538 were common at three time points, while 237, 1870 and 2448, were exclusively exhibited at 24 hpi, 48 hpi and 72 hpi, respectively (Fig. 2B). Furthermore, the heatmap of expression levels of all these melon DEGs showed that the expression profiles of them varied significantly in response to *P*. *xanthii* infection (Fig. 2C). A total of 1114 genes showed differential expression patterns at 24 hpi, from which 863 were found to be upregulated and 251 downregulated. The number of DEGs at 48 hpi was 3785, with 2781 upregulated and 1004 downregulated genes. At 72 hpi 4226 DEGs were detected. Of these, 2697 exhibited upregulation and 1529 showed downregulation (Fig. 2D).

### Gene functional enrichment analysis of DEGs and metabolism overview

To understand the primary functions of melon DEGs in response to *P*. *xanthii* infection, a gene functional enrichment analysis was performed using GENECODIS. A singular enrichment analysis of the GO biological process revealed a total of 33, 108 and 83 significantly enriched (adj. p-val. < 0.05) Gene Ontology (GO) terms at 24, 48 and 72 hpi, respectively (Table S2). These GO terms were ordered according to their hypergeometric *p-value* (Hyp), with the first positions being those with minor Hyp values. Subsequently, the GO terms were filtered to select the top 20 to reduce the amount of data and to facilitate the representation of the most enriched terms at each time point (Table 1). Among the first 20 GO terms over the three analysed time points, photosynthesis-related terms such as photosynthesis (GO:0015979), response to red light (GO:0010114), response to blue light (GO:0009637), response to far red light (GO:0010218) or response to light stimulus (GO:0009416) were common. Furthermore, GO terms for secondary metabolism-related genes such as response to wounding (GO:0009611), defence response to fungus (GO:0050832) or lignin biosynthesis process (GO:0009809) were also highly represented, with these GO terms being especially important at 72 hpi. In addition, other terms related to hormone signalling such as response to jasmonic acid stimulus (GO:0009753), response to abscisic acid stimulus (GO:0009757) or response to salicylic acid stimulus (GO:0009751) were also present as biological process functional enrichment.

**Table 1:** Top 20 GO biological terms for *P*. *xanthii*-infected melon leaves at 24, 48 and 72 hpi, as performed by the functional enrichment of all the DEGs in web-based GeneCodis software.

24 hpi	48 hpi	72 hpi
GO:0015979 photosynthesis	GO:0015979 photosynthesis	GO:0046686 response to cadmium ion
GO:0009409 response to cold	GO:0009414 response to water deprivation	GO:0008152 metabolic process
GO:0010114 response to red light	GO:0009611 response to wounding	GO:0015979 photosynthesis
GO:0009637 response to blue light	GO:0010114 response to red light	GO:0009611 response to wounding
GO:0046686 response to cadmium ion	GO:0009409 response to cold	GO:0006979 response to oxidative stress
GO:0010218 response to far red light	GO:0009651 response to salt stress	GO:0050832 defense response to fungus
GO:0009611 response to wounding	GO:0009753 response to jasmonic acid stimulus	GO0010114 response to red light
GO:0009416 response to light stimulus	GO:0009737 response to abscisic acid stimulus	GO:0009651 response to salt stress
GO:0019253 reductive pentose-phosphate cycle	GO:0006857 oligopeptide transport	GO:0080167 response to karrikin
GO:0015977 carbon fixation	GO:0010218 response to far red light	GO:0009737 response to abscisic acid stimulus
GO:0009744 response to sucrose stimulus	GO:0009637 response to blue light	GO:0010218 response to far red light
GO:0080167 response to karrikin	GO:0009416 response to light stimulus	GO:0055114 oxidation-reduction process
GO:0006979 response to oxidative stress	GO:0007623 circadian rhythm	GO:0019464 glycine decarboxylation via glycine cleavage system
GO:0009250 glucan biosynthetic process	GO:0080167 response to karrikin	GO:0009809 lignin biosynthetic process
GO:0009695 jasmonic acid biosynthetic process	GO:0055114 oxidation-reduction process	GO:0042128 nitrate assimilation
GO:0006857 oligopeptide transport	GO:0008152 metabolic process	GO:0009414 response to water deprivation
GO:0050832 defense response to fungus	GO:0019253 reductive pentose-phosphate cycle	GO:0010224 response to UV-B
GO:0006096 glycolysis	GO:0006096 glycolysis	GO:0009751 response to salicylic acid stimulus
GO:0009753 response to jasmonic acid stimulus	GO:0006970 response to osmotic stress	GO:0042742 defense response to bacterium
GO:0010105 negative regulation of ethylene mediated signaling pathway	GO:0015995 chlorophyll biosynthetic process	GO:0009637 response to blue light

**Figure 3:**
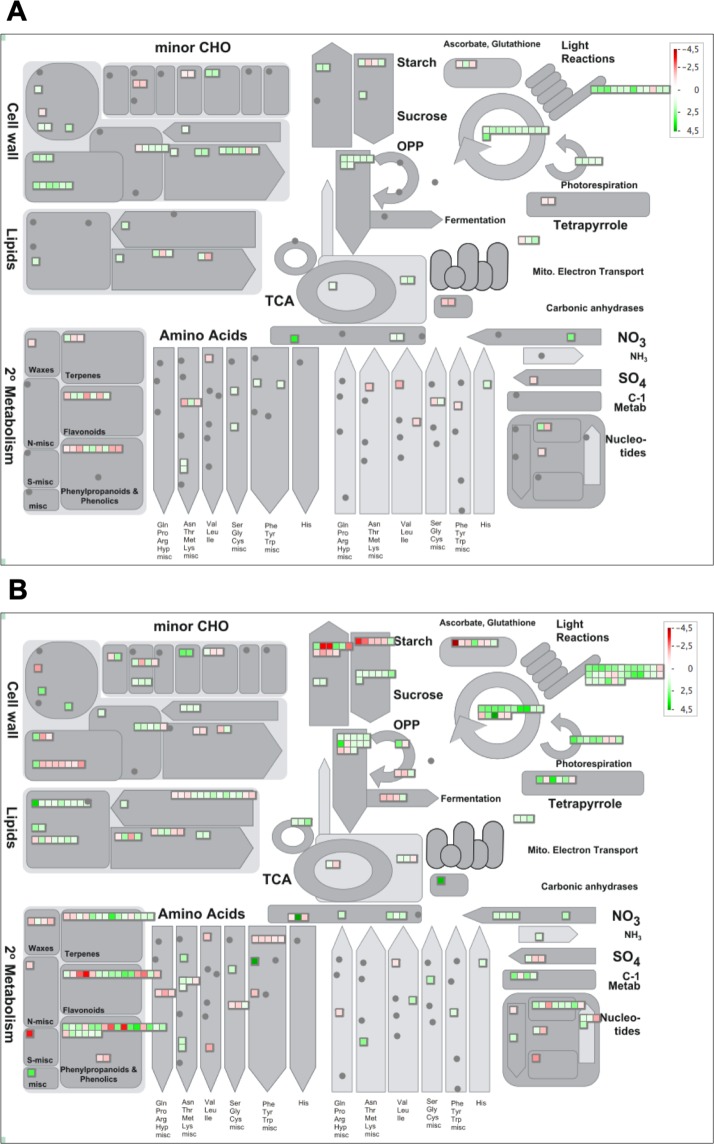
MapMan overview of plant metabolism in *P*. *xanthii*-infected melon leaves showing all the DEGs at 24 (**A**) and 72 hpi (**B**). The analysis was performed using MapMan v.3.5.0. Individual genes are represented by small squares. The colour key represents the RPKM (Reads Per Kilobase Million)-normalized log2-transformed counts. Red represents the downregulation and green represents the upregulation of melon genes from infected plants compared to uninfected controls.

Since the main dysregulated processes were similar at the three time points analyzed, to obtain a more comprehensive overview of the primary process dysregulation that occurred in melon plants during the first stages of *P*. *xanthii* infection, we focused our study at 24 and 72 hpi and performed a MapMan analysis of DEGs at those time points (Fig. 3). In general terms, at 24 hpi, primary metabolism-related genes such as light reaction genes, Calvin cycle genes and photorespiration genes were upregulated, whereas some secondary metabolism-related genes such as phenylpropanoid and flavonoid biosynthesis genes were slightly downregulated. Furthermore, cell wall modification-related genes were generally upregulated (Fig. 3A). At 72 hpi, the light reaction genes, Calvin cycle genes and photorespiration genes were also upregulated. Similarly, certain secondary metabolism related genes were downregulated, and a few of them were strongly repressed. Additionally, some starch synthesis and degradation-related genes were also down-regulated. However, compared to 24 hpi, the cell wall modification-related genes were mostly downregulated at 72 hpi (Fig. 3B). The MapMan analysis allowed us to validate that the most important GO terms were identified, completing the information and specifying the relative expression level of the primary dysregulated processes. Therefore, in general, photosynthesis and related processes were upregulated, whereas the major secondary metabolism pathways such us the phenylpropanoid biosynthesis pathway were downregulated.

### *P*. *xanthii* infection induces changes in the expression of melon genes involved in photosynthesis and secondary metabolism

**Figure 4:**
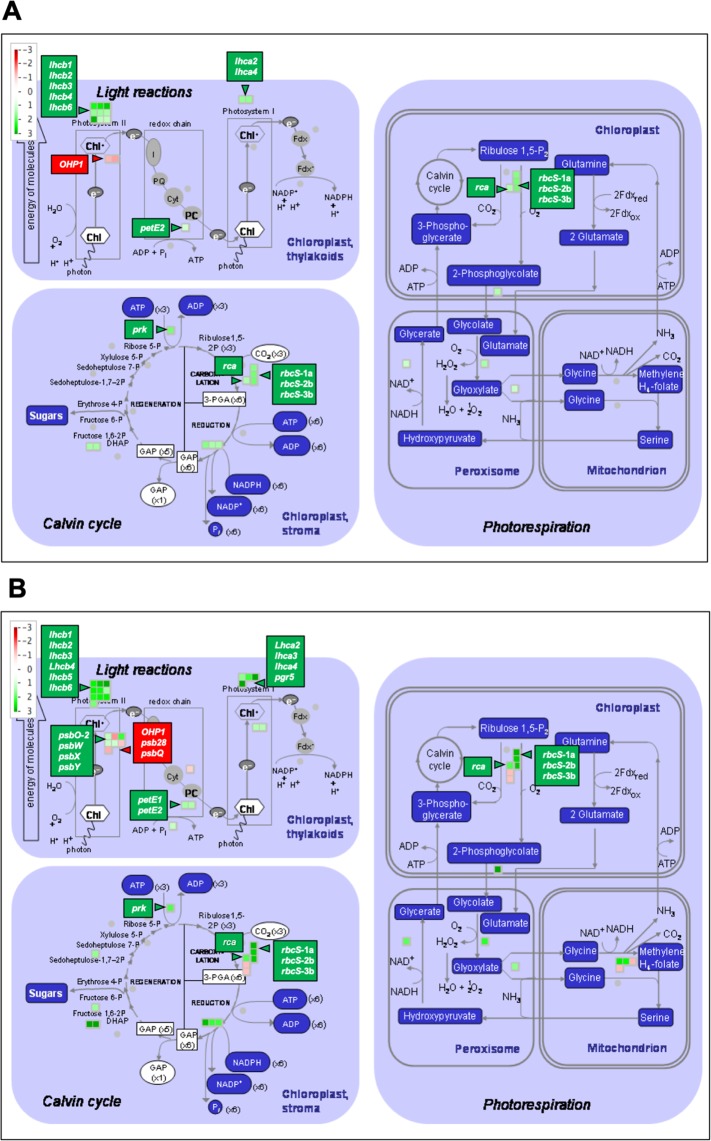
MapMan overview of primary photosynthetic metabolism in *P*. *xanthii*-infected melon leaves showing all the DEGs at 24 (**A**) and 72 hpi (**B**). The analysis was performed using MapMan v.3.5.0. Individual genes are represented by small squares. The colour key represents the RPKM-normalized log2-transformed counts. Red represents the downregulation and green represents the upregulation of melon genes from infected plants compared to uninfected controls.

To more thoroughly visualize the genes subjected to gene expression changes in some of the primary process that were dysregulated in melon plants infected by *P*. *xanthii*, a MapMan analyses specific for photosynthesis and secondary metabolism were performed at 24 and 72 hpi (Fig. 4). Among the 28 DEGs involved in photosynthesis and related processes at 24 hpi, 26 were upregulated, whereas only 2 were downregulated (Table S3). For example, several *lhc* genes that encode Lhcb and Lhca antenna proteins of PSII and photosystem I (PSI), respectively, or *petE2* that encodes the predominant plastocyanin isoform, were upregulated. In addition, some Calvin cycle genes such as those encoding ribulose bisphosphate carboxylase small chain (*rbcS-1a*, *rbcS-2b* and *rbcS-3b*), phosphoribulokinase (*prk*) and rubisco activase (*rca*) were also overexpressed. The only two suppressed genes were *psbT* and *OHP1*, which encode the PSII 5 kDa protein subunit PSII-T and one helix protein 1, respectively (Fig. 4A). At 72 hpi, 50 photosynthesis-related genes were DEGs. Forty-one of these genes were upregulated and 9 downregulated (Table S3). Among the upregulated genes, we note the other *lhc* genes (*lhcb5* and *lhca3*), *psb* genes of PSII (*psbO-*2, *psbW*, *psbX*, and *psbY*) or *petE1* and *pgr5*, codifying isoform 1 of plastocyanin and proton gradient regulation 5 protein, respectively. Although the *psbT* gene was not repressed, *psbQ* and *psb28* were downregulated at 72 hpi (Fig. 4B).

**Figure 5:**
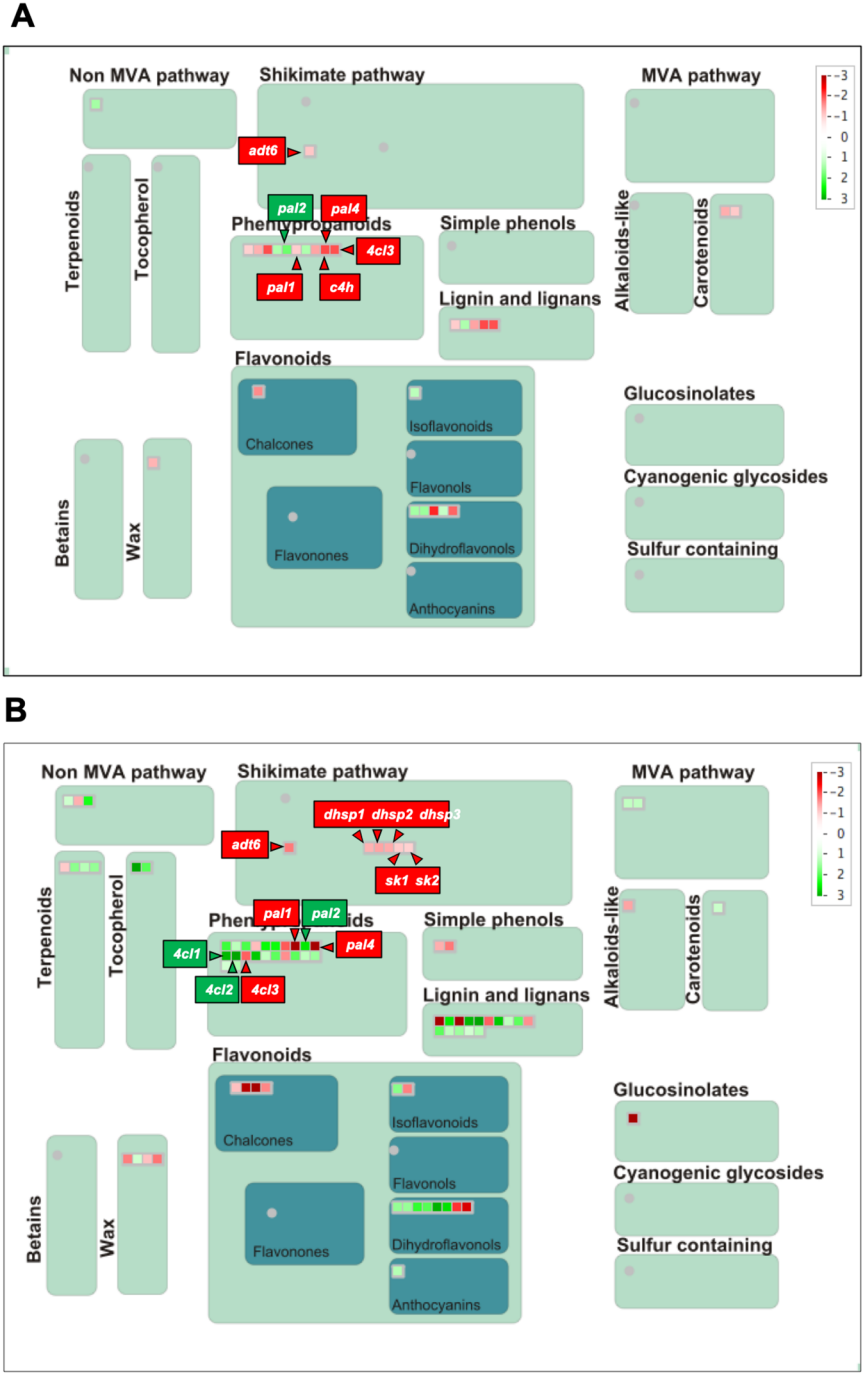
MapMan overview of secondary metabolism in *P*. *xanthii*-infected melon leaves showing all DEGs at 24 (**A**) and 72 hpi (**B**). The analysis was performed using MapMan v.3.5.0. Individual genes are represented by small squares. The colour key represents the RPKM-normalized log2-transformed counts. Red represents the downregulation and green represents the upregulation of melon genes from infected plants compared to uninfected controls.

A similar approach was performed to analyse the changes in the expression of secondary metabolism-related genes in *P*. *xanthii*-infected melon plants (Fig. 5). Among the 20 DEGs involved in secondary metabolism and detected at 24 hpi, 12 were downregulated and 8 were upregulated (Table S3). Several genes involved in the phenylpropanoid and shikimate pathways, which have a key role in the biosynthesis of different defence compounds against pathogens, were repressed, including the phenylalanine ammonia lyase isoform genes *pal1* and *pal4*, which are involved in the first step of phenylpropanoids biosynthesis. By contrast, *pal2* was upregulated. Other important genes in the phenylpropanoids pathway such as *c4h* and *4cl3* were downregulated. Moreover, *adt6*, a gene related to phenylalanine biosynthesis, was also downregulated (Fig. 5A). At 72 hpi, 62 genes involved in secondary metabolism were DEGs, of which 28 were downregulated and 34 upregulated (Table S3). For example, *pal4* was strongly repressed, whereas *pal2* was overexpressed. In addition, although *4cl3* remained downregulated, *4cl1* and *4cl2* were overexpressed. Furthermore, *adt6* experienced an increase repression level and other genes in the shikimate pathway that trigger phenylalanine biosynthesis, such as *dhsp1*, *dhsp2*, *dhsp3*, *sk1* and *sk2*, were downregulated (Fig. 5B). In summary, at 24 hpi, the phenylpropanoid pathway seems to be affected at the phenylalanine and p-coumarate biosynthesis levels, whereas at 72 hpi, the inhibition of this pathway is upstream, at the first steps of shikimate synthesis (Fig. S1).

**Figure 6:**
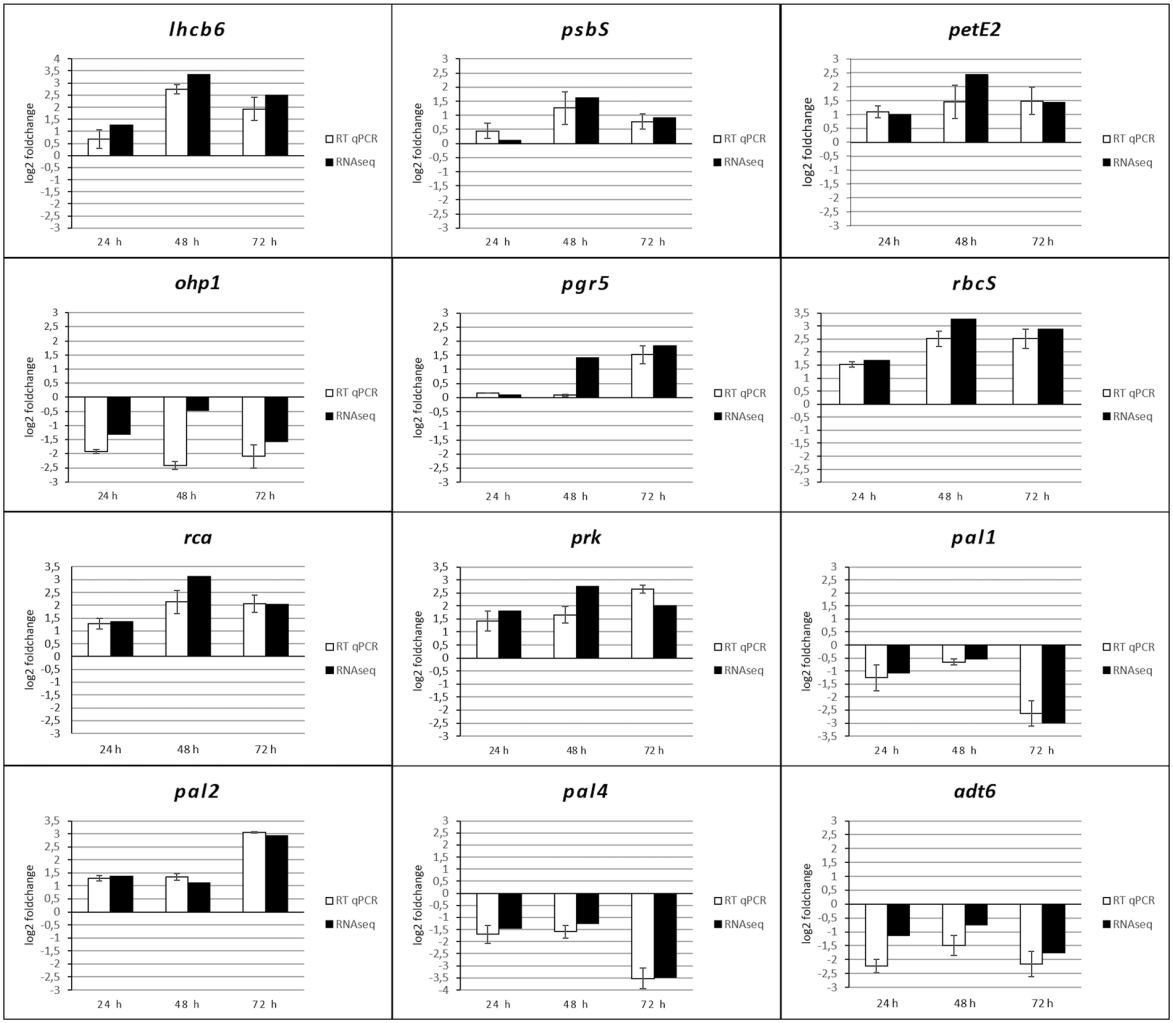
Validation of RNA-Seq data from *P*. *xanthii*-melon compatible interactions by qRT-PCR analysis of selected primary and secondary metabolism DEGs. The relative expression (log2-fold change) of twelve genes at 24, 48, and 72 hpi is shown. The transcript abundance was normalized to the transcription of the endogenous control β*-*actin gene (XM_008462689.2) and the relative expression of each gene was calibrated to the uninfected control at the corresponding time point. The qRT-PCR data (white bars) are expressed as the mean values of three experimental replicates from three independent experiments, with error bars depicting the standard error. The RNA-seq data (black bars) are also shown for comparison.

The results obtained by RNA-Seq were validated by qRT-PCR analysis. Twelve DEGs involved in photosynthesis and secondary metabolism were selected, subjected to expression analysis by qRT-PCR at the same time points and compared to the RNA-seq data. In general terms, the expression levels observed by qRT-PCR were very similar to those obtained by RNA-seq, with the only exceptions being the *OHP1* and *pgr5* at 48 hpi. These results allowed us to confirm the reliability of the RNA-seq data (Fig. 6).

### Fluorescence imaging analysis data from melon leaves in response to *P*. *xanthii* infection are consistent with the RNA-seq results

**Figure 7:**
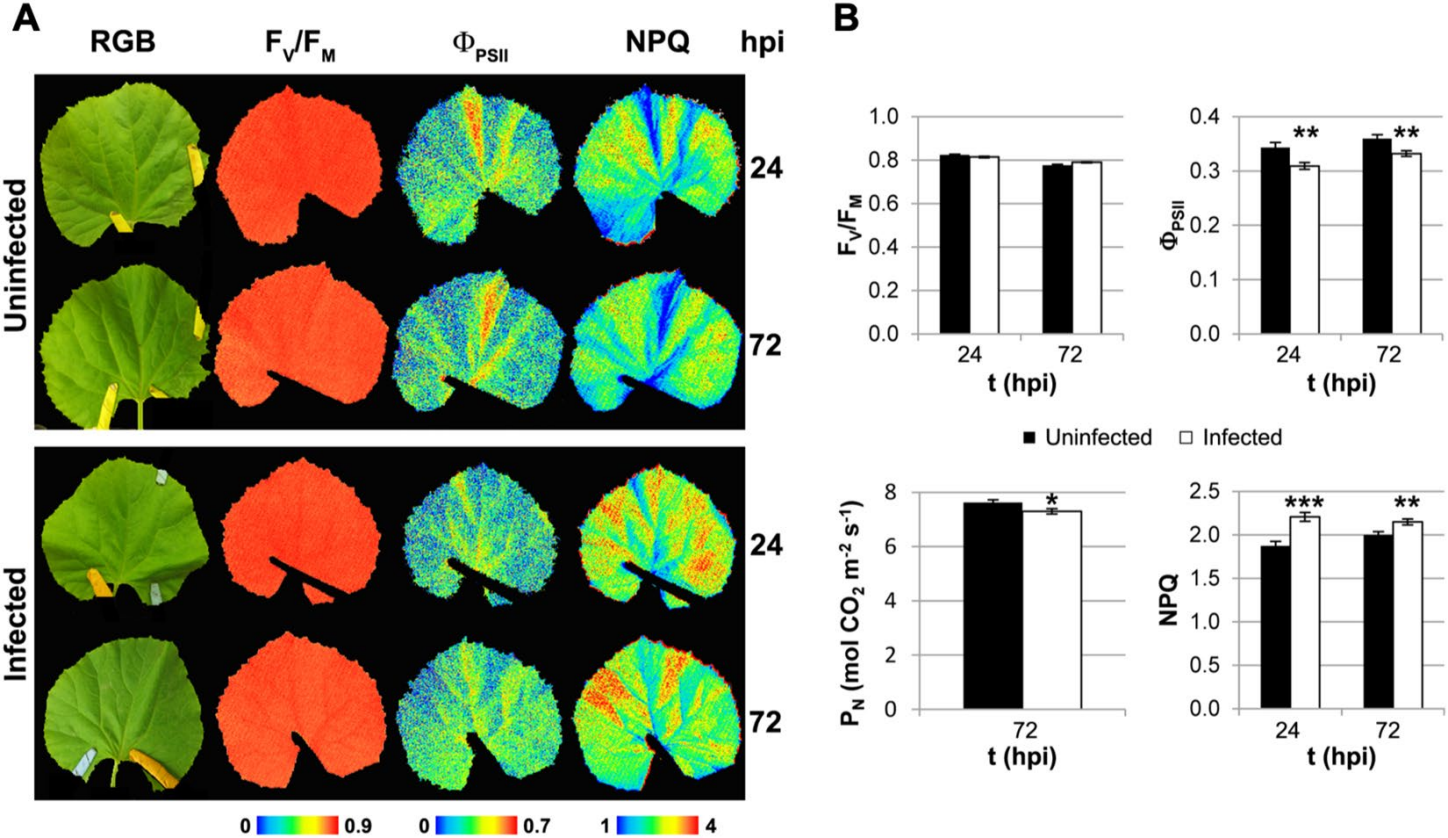
Impact of *P*. *xanthii* infection on the primary metabolism of melon leaves. (**A**) Standard images of the RGB, F_V_/F_M_, Φ_PSII_ and NPQ from non-infected and *P*. *xanthii*-infected melon leaves at 24 and 72 hpi. A false colour scale was applied for each parameter. Images from a representative experiment are shown. (**B**) The average values of F_V_/F_M_, Φ_PSII_, and NPQ (n = 6) and the net photosynthesis rate (n = 10) for non-infected and infected melon leaves at 24 and 72 hpi are shown, with bars representing the standard error. The asterisks indicate a statistically significant difference between the samples according to a Student’s t-test (^∗^P < 0.1, ^∗∗^P < 0.01, and ^∗∗∗^P < 0.001). The abbreviations are: F_V_/F_M_, maximum quantum yield of PSII; Φ_PSII_, effective quantum yield of PSII; NPQ, non-photochemical quenching; and P_N_, net photosynthesis rate.

To functionally validate the expression data revealed by RNA-seq analysis, the impact of the fungal infection on melon physiology was investigated by different fluorescence imaging techniques. The impact on leaf photosynthesis was investigated in terms of PSII efficiency and Calvin cycle activity (Fig. 7). The maximum quantum yield of PSII as measured as the F_V_/F_M_, did not show significant differences between infected and uninfected leaves. However, a consistent inhibition of the PSII (at 24 and 72 hpi) could be measured as a decrease in the Φ_PSII_ of infected leaves, correlating with a reduction in the net CO_2_ fixation rate (P_N_) of infected leaves at 72 hpi. Notably, the NPQ was significantly higher in infected leaves at 24 and 72 hpi. In addition, all these changes in the Chl-FI parameters could not be attributed to alterations in the contents of pigments such as chlorophylls, carotenoids or xanthophylls induced by the fungus, since no significant differences were found between infected and uninfected plants (Table S4).

**Figure 8:**
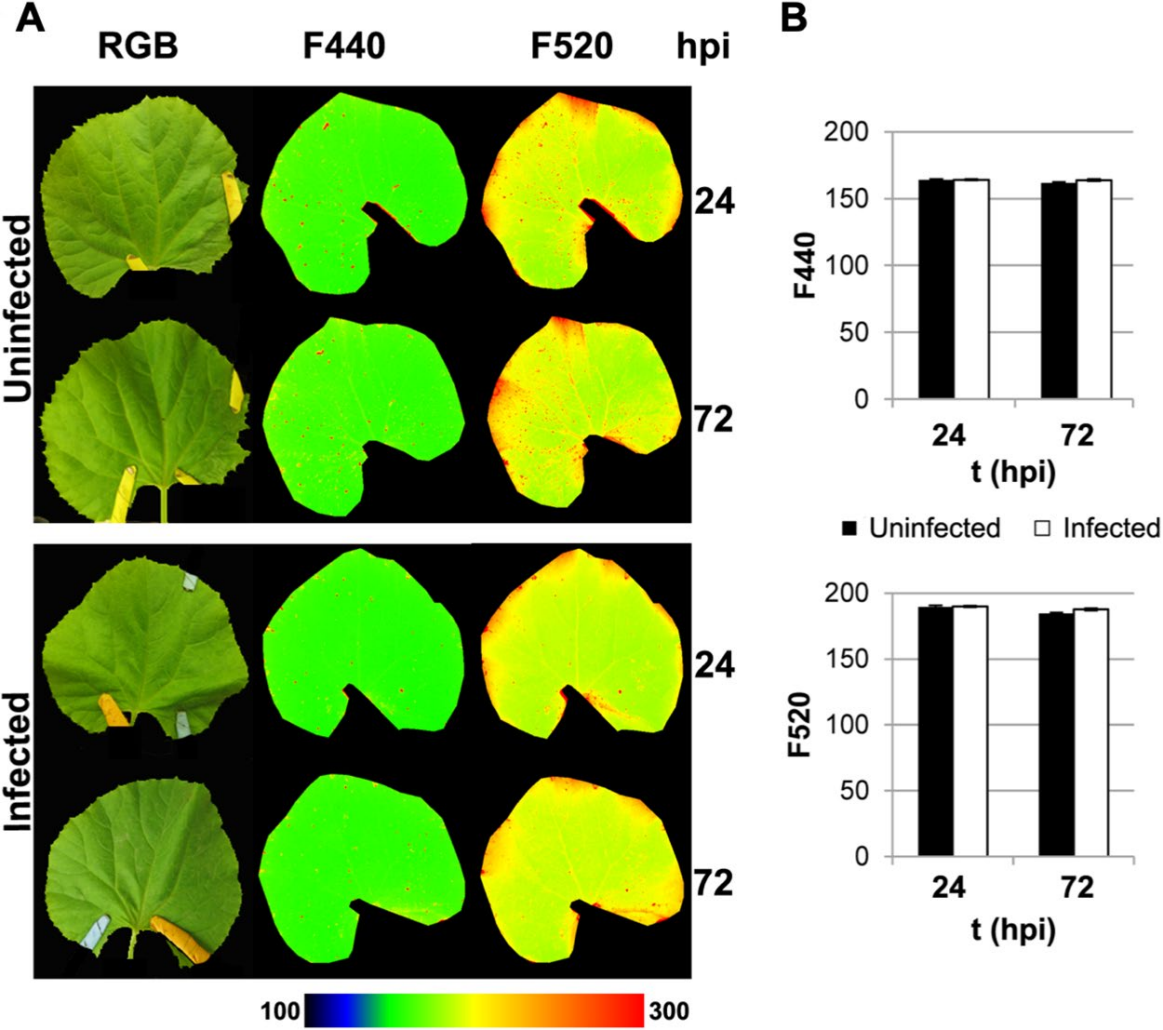
Impact of *P*. *xanthii* infection on the secondary metabolism of melon leaves. (**A**) Standard images of the RGB, F440 and F520 emitted by phenolic compounds from non-infected and *P*. *xanthii*-infected melon leaves at 24 and 72 hpi. A false colour scale was applied for each parameter. Images from a representative experiment are shown. (**B**) The average values of F440 and F520 (n = 6) for non-ninfected and infected melon leaves at 24 and 72 hpi are shown, with bars representing the standard error. No statistically significant differences between the samples were obtained according to a Student’s t test. The abbreviations are F440, blue fluorescence and F520, green fluorescence.

The accumulation of secondary metabolites in response to fungal infection was analysed by BGFI (Fig. 8). The F440 and F520 emitted by the infected leaves were comparable to that released by the uninfected controls at 24 and 72 hpi. This result indicated that there was no alteration in the accumulation of phenolic compounds in response to *P*. *xanthii* infection. In conclusion, and as revealed by RNA-seq analysis, this fungal infection seems to alter the photosynthesis efficiency of susceptible melon plants and manipulate the activation of the phenylpropanoid pathway.

## Discussion

The cucurbit powdery mildew disease elicited by *P*. *xanthii* is one of the most important limiting factors in cucurbit production worldwide^1^. The molecular mechanisms underlying this pathogenic interaction remain largely unknown, with only a few studies addressing the transcriptomic analysis in this fungus^9^ and in resistant pumpkin and melon lines^23,28^. For that reason, this study is intended to decipher the primary molecular and physiological changes taking place in melon plants during the first stages of the compatible interaction with *P*. *xanthii*. To this end, we analyzed this interaction using a combination of RNA-seq with different imaging techniques.

RNA-seq is a sensitive and effective approach for identifying the primary changes in gene expression^20,21^, but it generates a large amount of data that are difficult to process. To visualize the data and to perform a more efficient analysis, tools such as GO terms enrichment or MapMan analyses were used^23,36,37^. Although some terms obtained by GO enrichment analysis were different at the three time points analyzed here, most of them were related to two major physiological processes, photosynthesis and secondary metabolism. Therefore, to yield a simpler and comprehensive study, the subsequent analyses were performed using 24 and 72 hpi data points, which, on the other hand, were the time points from which image analysis data were obtained. The metabolism overview performed by MapMan yielded very similar results relative to the ones obtained through GO terms enrichment analysis, but the first approach allowed us obtain more visual results and to identify the primary genes dysregulated in each pathway. Therefore, by combining both analyses, it has been possible to detect different functional processes and related pathways that were being dysregulated in the melon plants infected with *P*. *xanthii*, with photosynthesis and secondary metabolism being the stand-outs.

Among all the photosynthesis-related DEGs, only *ohp1* was downregulated at both 24 and 72 hpi. The Ohp1 protein is required for PSII function^38^. Therefore, the downregulation of this gene could be part of a mechanism to decrease Φ_PSII_ in a regulated manner. However, *lhcb6* was upregulated upon inoculation at all the data points. Lhcb6 is a subunit of the minor light harvesting complex of PSII that is involved directly in the thermal dissipation of excess energy^39,40^. Furthermore, *psbS* was up-regulated at 48 hpi, coinciding with the maximum expression of *lhcb6*. The interaction of PsbS with the minor antenna, including Lhcb6, is crucial for the regulation of NPQ^41,42^. Moreover, the expression of *petE2 and pgr5* was also upregulated. Both genes encode key players in the cyclic electron flow around PSI, which dissipates the ΔpH across the thylakoid^43–45^, and it is essential for the photoprotection of the thylakoids^46^. These transcriptomics data are consistent with those of other plants infected with powdery mildew, in which several photosynthesis-related genes were found to be upregulated^23,47,48^.

The photosynthesis-related RNA-seq results were validated by different functional approaches such as chlorophyll fluorescence imaging (Chl-FI) and the net photosynthesis rate (P_N_). Compared to the control leaves, the *P*. *xanthii*-infected melon leaves showed photosynthesis inhibition at two levels, in terms of the electron transport rate (decrease in Φ_PSII_) and carbon fixation (decrease in P_N_) from 24 hpi onwards. Moreover, in the infected leaves, the mechanism of energy dissipation, as measured as the NPQ, was induced, although the F_V_/F_M_ was not affected. The photosynthesis was previously shown to be inhibited, as measured as a decrease in the Φ_PSII_ and P_N_, while the NPQ increased during the fungal infection^35,49^. These photosynthetic changes were not attributed to alterations in the chlorophyll contents of melon leaves. Those data are in accordance with previously obtained results in cucumber leaves infected with *P*. *xanthii*, which displayed a decrease in the Φ_PSII_ several days before a reduction in the chlorophyll content could be detected^50^. Hence, the changes in the Φ_PSII_ and NPQ could be caused by a modification in the expression of genes such as *ohp1*, *lhcb6* and *psbS*. Swarbrick *et al*.^51^ demonstrated downregulation in the expression of photosynthetic genes such as *rcbS* and Chl-*a*/*b*-binding proteins (*cab*) related to a decrease in the P_N_ of barley leaves infected by powdery mildew. However, during *P*. *xanthii*-melon interaction, there were increases in the expression levels of important photosynthetic genes such as *rbcS 1-a*, *2-b*, *3-b*, *rca* and *prk*. This finding could be the result of the manipulation of gene regulation by *P*. *xanthii* to increase the photosynthesis and photosynthates in melon plants for its own benefit; nevertheless, the plant effectively inhibited the photosynthetic activity, as a part of the plant defence programme to limit carbon source availability for the pathogen^52^. Cyclic electron flow around PSI, for which *petE* and *pgr5* genes were found to be upregulated, could contribute to the inhibition of the Calvin cycle.

It is well known that phenylpropanoid compounds play a key role in plant defence against pathogens^53^. Therefore, the repression of different DEGs related to this pathway and the shikimate pathway could be important for the establishment of compatible interactions. Whereas *c4h*, a key gene in lignin synthesis^54^, was downregulated at 24 hpi, did not show expression changes at 72 hpi, suggesting that it could be affecting the phenylalanine synthesis only at very early stages of the interaction. However *adt6*, *pal1*, *pal4* and *4cl3* were downregulated at 24 and 72 hpi. The arogenate pathway has been described as the predominant pathway for phenylalanine synthesis^55,56^, hence, enzymes such as prephenate aminotransferase (PAT) or arogenate dehydratase (ADT) become key action points. In relation to this situation, the observed downregulation of *adt6* could interfere with phenylalanine synthesis. The repression of *pal* genes could be even more important. This gene encodes enzymes that catalyse the first step in the phenylpropanoid pathway and are considered a key point of regulation between primary and secondary metabolism^57^. Between the *pal1* and *pal2* genes, Rohde *et al*.^58^ qualified *pal1* as being more important for phenylpropanoid biosynthesis. Moreover, different studies indicate that *pal1* expression is induced by wounding and pathogen attack^59–62^. In addition, *pal4* has been shown to act as an important gene in lignin biosynthesis^58,63,64^. In our study, both the *pal1* and *pal4* genes showed increased repression at 72 hpi, when the fungal development was more evident. The *4cl3* repression does not seem very important, since its function is related to flavonoid biosynthesis, but not to lignin biosynthesis, whose function is performed primarily by *4cl1* and *4cl2*^65^, both of which are upregulated at 72 hpi. However, the repression of several genes in the shikimate pathway at 72 hpi, such as *dahps1*, *dahps2*, *dahps3*, *sk1* and *sk2*, was more important since they act as shikimate pathway regulators^65^. The *dahps* (3-Deoxy-D-arabinoheptulosonate 7-phosphate synthase) genes act as a branch point to convert primary carbon source into the shikimate pathway and could act as a shikimate pathway key point to compete for phosphoenolpyruvate and erythrose 4-phosphate with the glycolysis and the pentose phosphate pathway^65^. Moreover, *dahps* genes are induced by wounding and fungal elicitors, which induce the synthesis of phenylpropanoids metabolites^66,67^. On other hand, several studies have suggested that *sk* genes have an important role in plant defense response, increasing their expression in the presence of fungal elicitors^68^. The repression of key genes related directly to plant defense such as *pal* or, *dahps* and *sk* when the development of *P*. *xanthii* increases, suggests that they are manipulated by the fungus as a strategy to favour infection development. Our RNA-seq data suggest that the phenylpropanoid synthesis is not totally blocked but is affected enough to avoid the accumulation of compounds necessary for plant defense, such as lignin or phytoalexins, resulting in a compatible interaction as described in other pathosystems^24,69^. Similarly, secondary metabolism-related genes have been found to be commonly repressed in other plant-powdery mildew compatible interactions^26,48,70^.

BGF experiments were conducted to validate the RNA-seq results on secondary metabolism. The accumulation of plant defence compounds is frequently found in diseased plants. This accumulation of host secondary metabolites could be measured as an increase in F440 and F550 in bacterial, viral and fungal infections^29^. However, in *P*. *xanthii* infection, the F440 and F550 parameters did not display significant differences between uninfected and infected melon leaves, pointing to no changes in the accumulation of secondary metabolites in response to *P*. *xanthii*. This result is in accordance with a previous study on the barley powdery mildew *Blumeria graminis* in susceptible barley plants^51^. Furthermore, *P*. *xanthii* infection in zucchini plants was related to an increase of blue-green fluorescence from the leaves^71^. However, in that case, the fluorescence was shown to be emitted by components of the fungal cell walls and not by plant secondary metabolites.

But what is the situation in cucurbit cultivars resistant to *P*. *xanthii*? Two transcriptomic studies carried out with pumpkin and melon lines resistant to *P*. *xanthii* showed that different genes of photosynthesis and secondary metabolism were differentially expressed^23,28^. Thus, most of photosynthesis related genes such as *psb27*, *psbP*, *psaK*, *psaE*, *psaH*, *psaG* or *psaN* were up-regulated in the pumpkin inbred resistant line “112-2”^23^, whereas in the melon resistant cultivar MR-1, carbon fixation related genes were up-regulated at the early stages of the interaction, but they were down-regulated at 72 hpi^28^. However, in the melon susceptible cultivar “Rochet” examined in this work, other photosynthesis related genes were either down-regulated such as *ohp1* or up-regulated such as *lhcb6*, *petE* and *pgr5*. In addition, the carbon fixation related genes were up-regulated at all time points analyzed. With respect to the secondary metabolism related genes, in the pumpkin resistant line *4cl3* or *adt6* were up-regulated^23^. Aligned to this, in the melon resistant cultivar most of the pathogen-induced DEGs were enriched in pathways related to flavonoids, phytoalexins and phenylpropanoid biosynthesis, such as, for example, the *pal* genes that increased their expression after exposure to the pathogen^28^. In contrast, in the melon susceptible cultivar, most of the secondary metabolism genes were down-regulated, including the *pal* genes. In other words, the genes of photosynthesis and secondary metabolism of susceptible and resistant plants are expressed differentially and in opposite ways in response to the attack of *P*. *xanthii*.

Our RNA-seq and fluorescence imaging results suggest that during the first stages of infection, *P*. *xanthii* manipulates plant gene expression. Because of the plant-pathogen interaction, melon leaf physiology and especially photosynthesis and secondary metabolism were altered. Host manipulation is believed to be carried out by the panel of effectors secreted by the pathogen^72^. During host colonization, *P*. *xanthii* secretes an important number of proteins, most of which have an unknown function^9,11^. We believe that some of these proteins could be responsible for reprogramming the melon physiology to allow disease establishment. The catalogues of *P*. *xanthii* effector candidates, both epiphytic^9^ and haustorial (Polonio *et al*., unpublished results), are known. To identify *P*. *xanthii* effectors that specifically target photosynthesis or secondary metabolism, different approaches such as yeast two-hybrid screening^73–75^ or dual RNA-seq analysis^76,77^ can be undertaken. Currently, we are resolving the melon-*P*. *xanthii* interactions through a dual RNA-seq approach.

## Methods

### Growth conditions of plants and fungi

Melon plants (*Cucumis melo* L.) cv Rochet (Semillas Fitó, Barcelona, Spain) susceptible to the *P*. *xanthii* isolate 2086 were used in all the experiments. The plants were cultivated in a growth chamber with a 16 h: 8 h, light: dark cycle at 25 °C for one month. The powdery mildew isolate was cultured on previously disinfected zucchini (*Cucurbita pepo* L.) cotyledons cv Negro Belleza (Semillas Fitó) and maintained in Bertrand medium under a 16 h: 8 h, light: dark cycle at 22 °C for one week^78^.

### Experimental design

To elucidate the changes in the gene expression levels in these melon plants during the first stages of their compatible interactions with *P*. *xanthii*, we performed an RNA-seq analysis at 24 h, 48 h and 72 h post-inoculation (hpi). For the inoculation, *P*. *xanthii* conidia were collected by immersing infected zucchini cotyledons in 50 ml of a 0.01% Tween-20/distilled water solution. The third and fourth true leaves of 1-month-old melon plants were inoculated with a spore suspension at 1 × 10^6^ conidia ml^−1^ (infected plants) or with 0.01% Tween-20 in distilled water (control plants). We used the reference transcriptome of *C*. *melo* to define the differentially expressed genes (DEGs) and later orthologous annotation with *Arabidopsis thaliana* to perform GO enrichment and MapMan analysis. In parallel, the changes in the physiology of the melon plants induced by the infection were analysed using different imaging techniques and other experimental approaches as described below.

### Confocal laser scanning microscopy (CLSM)

To visualize the development of the fungal structures during the initial stages of the infection process, a CLSM analysis was performed. For this purpose, leaf discs were taken at the same time points selected for RNA-seq analysis and the fungal structures were stained with an aqueous solution of 100 µg mL^−1^ propidium iodide^79^. The samples were observed using a Leica SP5 II confocal microscope (Leica Microsystems, Wetzlar, Germany). The samples were excited with a 488 nm laser line and their fluorescence was detected over a 510–570 bandpass range. Bright field images were taken using the transmission channel. All the images were observed using a 40× oil-immersion objective and were processed with Leica LAS AF software (LCS Lite, Leica Microsystems).

### RNA isolation, cDNA library construction and Illumina sequencing

For RNA isolation, melon leaves were collected, immediately frozen in liquid nitrogen and stored at −80 °C until use. The frozen leaves were ground with a mortar and pestle and the total RNA was extracted using TRI Reagent (Sigma-Aldrich, Saint Louis, USA) according to the manufacturer’s instructions. The total RNA was quantified using a NanoDrop 2000 spectrophotometer (Thermo Fisher Scientific, Waltham, MA, USA). The quality and quantity of the RNA were measured on an Agilent Bioanalyzer 2100 using an RNA Pico 6000 chip (Agilent Technologies, Santa Clara, CA, USA). Approximately 1 μg of each sample was used for cDNA library construction using an Illumina Stranded mRNA Sample Preparation Kit (Illumina, San Diego, CA, USA) according to the manufacturer’s instructions. Finally, the cDNA libraries were sequenced by Illumina NextSeq 550 system (Illumina).

### RNA-Seq data analysis

The detailed strategy for the differential expression analysis is depicted in Fig. S2. The analysis process was automatized using Autoflow^80^. The raw reads were pre-processed using the SeqTrimNext pipeline^81^ (http://www.scbi.uma.es/seqtrimnext) available at the Plataforma Andaluza de Bioinformática (University of Málaga, Spain) using the specific NGS technology configuration parameters. This pre-processing removes low quality, ambiguous and low complexity stretches, linkers, adaptors, vector fragments, organelle DNA, polyA, polyT tails, and contaminated sequences while keeping the longest informative part of the read. SeqTrimNext also discarded sequences below 25 bp. Subsequently, the clean reads were aligned with the melon transcriptome sequence^82^ (http://melonomics.cragenomica.es/files/Transcriptome/) with Bowtie2^83^ in BAM files, which were then sorted and indexed using SAMtools v1.4^84^. The reads containing discordant alignments were rejected due to their ambiguous location. Uniquely localized reads were used to calculate the read number value for each gene. Differentially expressed genes (DEGs) between two samples were analyzed using DESeq. 2^85^, one of the R packages. For each gene, a P-value < 0.05 and log2-fold change >1 or < −1 were considered the significance threshold. All the DEGs were annotated with orthologous genes in *A*. *thaliana* (https://plants.ensembl.org/Arabidopsis_thaliana/Info/Index) using Full-LengtherNext (http://www.scbi.uma.es/fulllengthernext) and they were hierarchichally clustered by heatmap representation.

### Gene functional enrichment and MapMan analysis of DEGs

The DEGs annotated with orthologous genes in *A*. *thaliana* were used to identify the Gene Ontology functional categories using web-based GENECODIS software^86–88^ (http://genecodis.cnb.csic.es/). MapMan software v. 3.5.0^89^ was used to provide a graphical overview of the metabolic and regulatory pathways for the detected DEGs.

### Quantitative reverse transcription (qRT)-PCR

The gene expression for the selected plant genes was quantified by qRT-PCR. The total RNA was isolated from the melon plants as described above. First-strand cDNA synthesis was performed using Invitrogen Superscript III Reverse Transcriptase (Invitrogen, Carlsbad, CA, USA) with random primers according to the manufacturer’s instructions. The qRT-PCR reactions were conducted in a CFX384 Touch Real-Time PCR detection system (Bio-Rad, Hercules, CA, USA) using SsoFast EvaGreen Supermix according to the manufacturer’s recommendations (Bio-Rad). Gene-specific primers (Table S5) were designed using Primer3^90^. The *C*. *melo* β*-*actin gene (XM_008462689.2) was used as a reference gene^91^. The qRT-PCR conditions were as follows: enzyme activation step at 95 °C for 30 s, followed by 40 cycles of 5 s at 95 °C and 5 s at 55 °C. After the amplifications, the data were analysed using CFX Manager Software (Bio-Rad). Additionally, the amplicon sizes were confirmed by visualization on 1% agarose gels.

### Analysis of photosynthetic activity

The photosynthetic performance in terms of PSII activity was studied by variable Chl-FI, using an Open FluorCam 700 MF (Photon System Instruments, Brno, Czechia). The applied protocol was the one described as number 1 by Pineda *et al*.^92^. Black and white images corresponding to transient chlorophyll fluorescence values were collected and used to calculate several fluorescence parameters, using FluorCam software version 5.0. The images obtained for these parameters (F_V_/F_M_: maximum quantum yield of PSII, Φ_PSII_: effective quantum yield of PSII and NPQ: non-photochemical quenching), were calculated according to Maxwell & Johnson^93^. A false colour scale was applied to the calculated images using the Fluorcam software.

The net photosynthesis rate (P_N_) was determined using an infrared gas analyser (IRGA LI-6400, Li-Cor Inc., Lincoln, NB, USA) on a 100 mm^2^ melon leaf area. For the measurements, radiation was supplied by a Qbeam solid state LED lighting system attached to the leaf cuvette (6400-02B LED, Li-Cor Inc.).

### Pigment content quantification

The pigment content was determined spectroscopically according to Lichtenthaler and Buschmann^94^. For each treatment, three leaf disks of 78 mm^2^ each were isolated and immediately frozen under liquid nitrogen. Quantifications were perfomed on acetone 80% (v/v in water) leaf extracts prepared by the homogenization of the tissue in liquid nitrogen and a subsequent centrifugation at 16000 g to remove the insoluble material. The absorbance at 470, 647 and 663 nm was measured using 80% acetone as a blank. The chlorophyll *a* (Chl *a*), chlorophyll *b* (Chl *b*), total chlorophyll (Chl T), and total xanthophyll and carotenoid (Xanth + Car) contents were determined according to the equations described in Lichtenthaler and Buschmann^94^.

### Multicolour fluorescence imaging (MCFI)

To further investigate the plant health status, and with special interest in plant secondary metabolism, the multicolour fluorescence emission from the adaxial side of melon leaves was recorded using an Open FluorCam FC 800-O (Photon Systems Instruments), according to Pérez-Bueno *et al*.^95^. Images of F680, F740 and particularly, BGF (F440 and F520 emitted by phenolic compounds from the phenylpropanoid pathway) from both uninfected and *P*. *xanthii*-infected melon leaves were captured by FluorCam software version 7.1.0.3, which also applied a false colour scale to the black and white images recorded here.

## Supplementary information

Supplementary figures and tables

Table S3

Table S4

## Data Availability

The complete RNA-seq sequencing data for all the samples were deposited in the NCBI Sequence Read Archive and are accessible under the accession number PRJNA434538.
