# Supplementary material for: RNA-seq analysis and fluorescence imaging of melon powdery mildew disease reveal an orchestrated reprogramming of host physiology
Source: Sci Rep. 2019 May 28;9:7978. doi: 10.1038/s41598-019-44443-5 (PMC6538759; doi:10.1038/s41598-019-44443-5)
Supplement: Supplementary file 1 — Supplementary figures and tables [file 41598_2019_44443_MOESM1_ESM.pdf]

Title

RNA-seq analysis fluorescence imaging of melon powdery mildew disease reveal an orchestrate reprogramming of host physiology

Authors:

Álvaro Polonio[1,2], polonio@uma.es

Mónica Pineda[3], mpineda@eez.csic.es

Rocío Bautista[4], rociobm@uma.es

Jesús Martínez-Cruz[1,2], jesusmcruz@uma.es

María Luisa Pérez-Bueno[3], marisa.perez@eez.csic.es

Matilde Barón[3], mbaron@eez.csic.es

Alejandro Pérez-García[1,2], aperez@uma.es

Table S1 Summary of statistics of RNA-Seq analysis of melon-*P. xanthii* interaction

Samples	Input reads	Output reads	Rejected reads (%)	Reads aligned to reference (%)
Uninfected24hpi_1	87,809,876	75,500,680	10.41	85.69
Uninfected24hpi_2	76,542,470	68,370,412	7.76	84.78
Uninfected24hpi_3	106,356,748	94,607,324	7.82	83.35
Uninfected48hpi_1	118,995,292	102,632,628	10.44	85.27
Uninfected48hpi_2	77,108,192	68,918,670	7.77	88.15
Uninfected48hpi_3	115,508,352	102702870	7.65	85.83
Uninfected72hpi_1	93,416,186	82,948,230	8.00	87.68
Uninfected72hpi_2	69,539,512	58,948,720	11.34	85.00
Uninfected72hpi_3	110,202,904	85,023,518	19.62	84.25
Infected24hpi_1	109,377,644	97,468,648	7.38	83.61
Infected24hpi_2	93,731,880	85,484,974	5.80	84.10
Infected24hpi_3	76,648,932	67,916,294	8.26	84.90
Infected48hpi_1	87,981,974	74,466,522	11.31	84.00
Infected48hpi_2	89,057,812	76,561,898	9.84	83.87
Infected48hpi_3	85,438,210	73,423,600	10.31	83.64
Infected72hpi_1	88,500,998	74,541,194	11.80	85.10
Infected72hpi_2	76,639,666	66,055,082	9.75	84.89
Infected72hpi_3	98,931,690	80,396,216	14.68	83.80

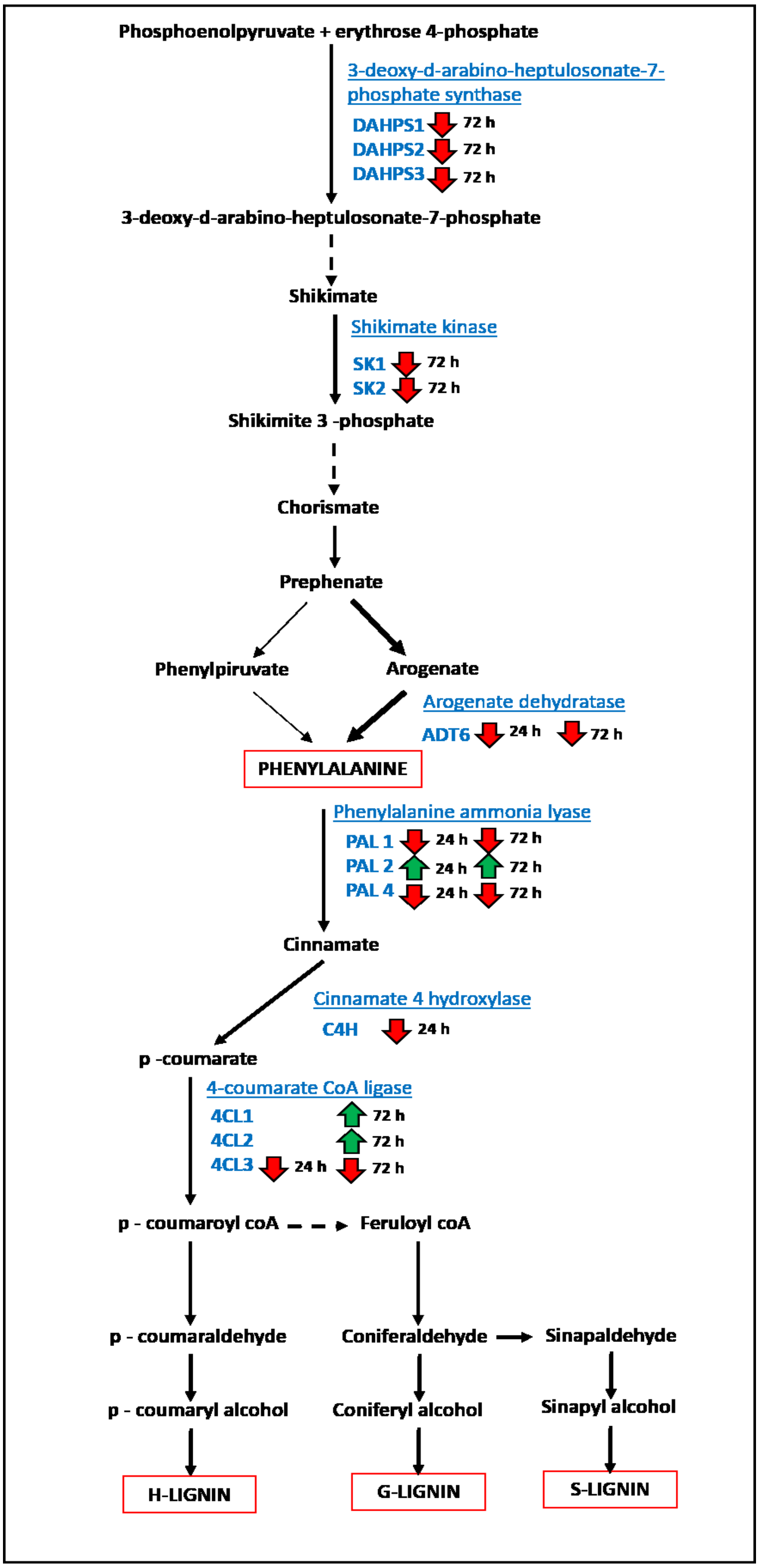

Fig. S1 Schematic representation of phenylpropanoids pathway of *P. xanthii*-infected melon leaves showing all DEGs at 24 and 72 hpi. The colour key represents dysregulation of melon genes from infected plants compared to uninfected controls. Green arrows represent up-regulated genes and red arrows represent down-regulated genes. Discontinuous black arrows show incomplete pathway

Table S4 Impact of *P. xanthii* infection on pigment content of melon leaves

Pigments[a]	24 hpi		72 hpi	
	Uninfected	Infected	Uninfected	Infected
Chl *a*	0.84±0.12[b,c]	0.78±0.03	0.73±0.04	0.65±0.05
Chl *b*	0.26±0.03	0.29±0.03	0.27±0.04	0.20±0.04
Chl T	1.10±0.12	1.08±0.06	1.00±0.09	0.85±0.09
Xant + Car	0.21±0.02	0.20±0.01	0.18±0.01	0.19±0.01

[a]Abbreviations are: Chl *a*, chlorophyll *a*; Chl *b*, chlorophyll *b*; Chl T, total chlorophyll content; Xant + Car, xanthophylls and carotenoids.

[b]Values shown are average values (n=6) in µg mg^{-1} of fresh weight ±SE.

[c]No statistically significant differences between uninfected and infected samples were obtained according to two-tailed Student's t test.

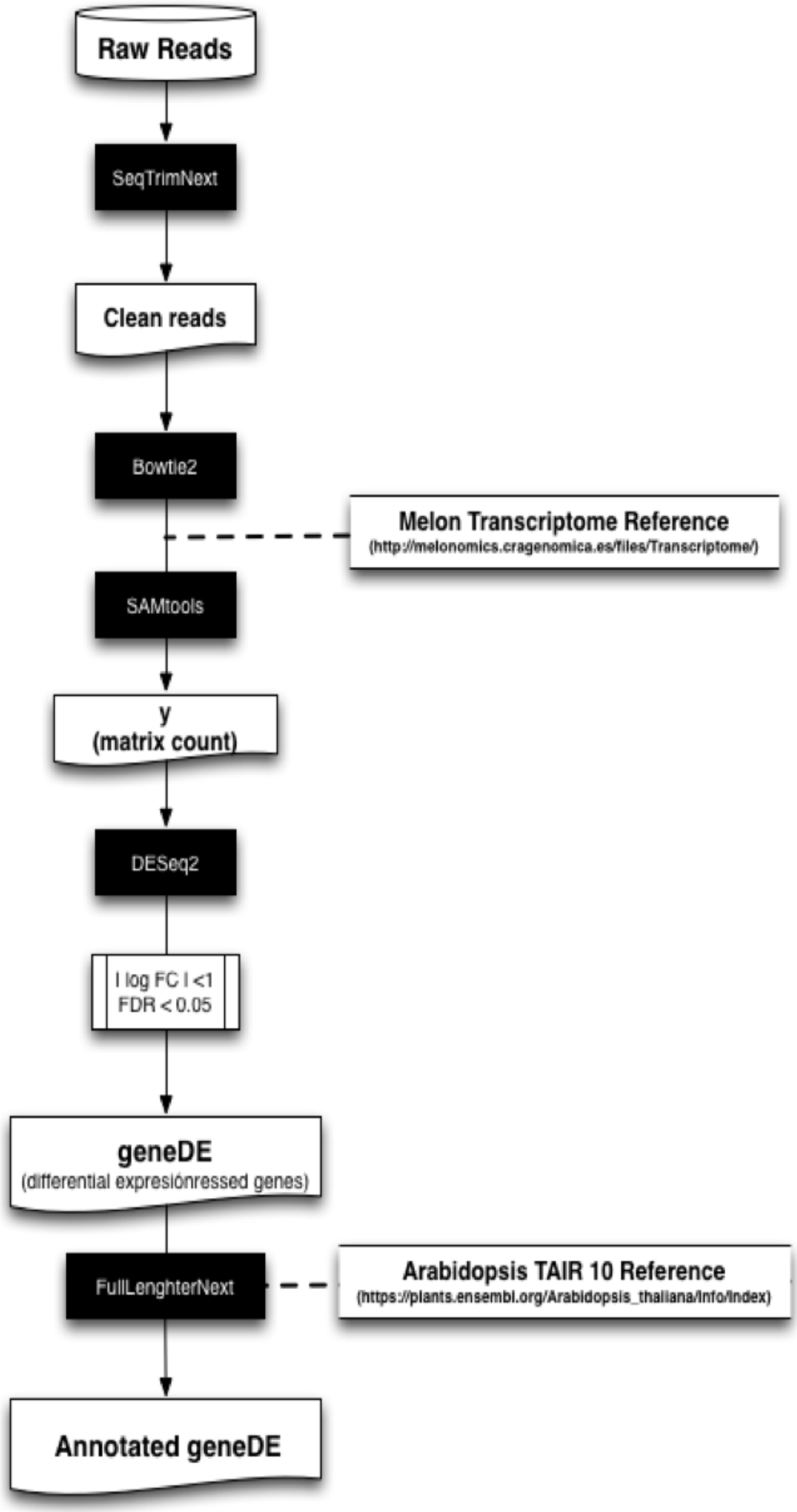

Fig S2. Schematic workflow used to perform the RNA-seq analysis of *P. xanthii*-melon compatible interaction

Table S5 Oligonucleotides used in this study

Gene	Primer name	Sequence	Amplicon size
lhcb6	lhcb6-F	5’-GAATCCCATGGTTCGAAGCC-3’	214 bp
	lhcb6-R	5’-GGATACCCTTGTTCTCCGGT-3’	
psbA	psbA-F	5’-TTCTGCAGCTATCGGTTTGC-3’	175 bp
	psbA-R	5’-AATCCAAGGACGCATACCCA-3’	
psbS	Psbs-F	5’-GTTTCACCCCTCTTGCTGTG-3’	175 bp
	Psbs-R	5’-AATGATGCAGCAAAGCCGAT-3’	
petE	petE2-F	5’-CATCCCCAAAGCTTAGCGTC-3’	201 bp
	petE2-R	5’-ATCCCGCGTTGTTCTTGAAC-3’	
gr5	pgr5-F	5’-CCTCTTTCCTCCTCCTTCCA-3’	176 pb
	pgr5-R	5’-GCAAGAACAACAACAGGTGC-3’	
rbcS	rbcS-2b-F	5’-GGGGAAGAGTTCAGTGCATG-3’	222 pb
	rbcS-2b-R	5’-ATGGTCCAGTAGCGTCCATC-3’	
pal1	pal1-F	5’-CAAGATCGCTATGCCCTTCG-3’	164 pb
	pal1-R	5’-CCTTGGAAGTTGCCTCCATG-3’	
pal2	pal2-F	5’-GCTGAGCAACACAACCAAGA-3’	231 pb
	pal2-R	5’-TGAAGGATCGAGTGCACCAT-3’	
pal4	pal4-F	5’-ACCACAATGTCACACCTTGC-3’	236 pb
	pal4-R	5’-TCCAACACCCGTCCCATTAA-3’	
rca	rca-F	5’-TGCTGGACTTCGACAATACG-3’	150 pb
	rca-R	5’-CCTCCCCAAATACCCAGAAT-3’	
prk	prk-F	5’-AACCCATGGCTGATAACCTG-3’	152 pb
	prk-R	5’-TCTGGATTTCCACCTCTTGG-3’	
ohp	ohp-F	5’-GGGGAAGAGGGAAAGTTCTG-3’	176 pb
	ohp-R	5’-GGCAGAAGACCCAATTTGAA-3’	
adt6	adt6-F	5’-ATCCATAACTCCACCGCTTG-3’	177 pb
	adt6-R	5’-GCCACTCACGATTACCGATT-3’	
